# Cross-Cultural Adaptation and Validation of the MPAM-R to Brazilian Portuguese and Proposal of a New Method to Calculate Factor Scores

**DOI:** 10.3389/fpsyg.2017.00261

**Published:** 2017-02-28

**Authors:** Maicon R. Albuquerque, Mariana C. Lopes, Jonas J. de Paula, Larissa O. Faria, Eveline T. Pereira, Varley T. da Costa

**Affiliations:** ^1^Department of Sports, Universidade Federal de Minas GeraisBelo Horizonte, Brazil; ^2^Department of Physical Education, Universidade Federal de ViçosaViçosa, Brazil; ^3^Department of Psychology, Faculdade de Ciências MédicasBelo Horizonte, Brazil

**Keywords:** motives, physical activity, factor score, motivation, psychometry

## Abstract

In order to understand the reasons that lead individuals to practice physical activity, researchers developed the Motives for Physical Activity Measure-Revised (MPAM-R) scale. In 2010, a translation of MPAM-R to Portuguese and its validation was performed. However, psychometric measures were not acceptable. In addition, factor scores in some sports psychology scales are calculated by the mean of scores by items of the factor. Nevertheless, it seems appropriate that items with higher factor loadings, extracted by Factor Analysis, have greater weight in the factor score, as items with lower factor loadings have less weight in the factor score. The aims of the present study are to translate, validate the MPAM-R for Portuguese versions, and investigate agreement between two methods used to calculate factor scores. Three hundred volunteers who were involved in physical activity programs for at least 6 months were collected. Confirmatory Factor Analysis of the 30 items indicated that the version did not fit the model. After excluding four items, the final model with 26 items showed acceptable model fit measures by Exploratory Factor Analysis, as well as it conceptually supports the five factors as the original proposal. When two methods are compared to calculate factors scores, our results showed that only “Enjoyment” and “Appearance” factors showed agreement between methods to calculate factor scores. So, the Portuguese version of the MPAM-R can be used in a Brazilian context, and a new proposal for the calculation of the factor score seems to be promising.

## Introduction

Physical activity is a well-documented method to reduce a number of diseases such as cardiovascular disease (Carnethon, 2009) and a widening variety of chronic diseases, including diabetes mellitus (Colberg et al., 2010; Burr et al., 2012b), cancer (Shi et al., 2015), obesity (Lakka and Bouchard, 2005), hypertension (Diaz and Shimbo, 2013), bone and joint diseases (Burr et al., 2012a). Physical activity is also a protective factor or useful intervention to reduce psychiatric symptoms in cognitive disorders (e.g., depression) (Lee et al., 2012) and improve cognitive functioning (Bamidis et al., 2014). Individuals of all ages, including healthy subjects, can achieve a number of benefits in different dimensions (physical, psychological, social, and emotional) through physical activity (Penedo and Dahn, 2005; Warburton et al., 2006; Vogel et al., 2009; Janssen and Leblanc, 2010). Nevertheless, Hallal et al. (2012) suggest that approximately 31% of adults and 80% of adolescents worldwide do not reach recommended levels of daily physical activity. Moreover, studies such as Lee et al. (2012) demonstrated that if inactivity decreased by 10%, half a million deaths could be prevented every year. Therefore, it seems crucial to understanding why some people are active and others are not.

Although the underlying causes of why some people are active and others not are highly complex, motivation is a possible explanatory factor. Motivation is a key process that influences individuals’ initiation and maintenance of behavior (Murcia et al., 2007a). The Self-Determination Theory (SDT) seems to be the most contemporary framework used to understand physical activity motivation and adherence (Hagger and Chatzisarantis, 2008; Molanorouzi et al., 2015). Based on SDT, motivation to engage and adherence to physical activity need to be distinguished, and the most basic distinction is between intrinsic and extrinsic motivation (Ryan and Deci, 2000a). Intrinsic motivation refers to doing something because it is inherently interesting or enjoyable (Ryan and Deci, 2000a,b). For instance, a Taekwondo practitioner who practices for enjoyment and challenge involved in the sport is said to be intrinsically motivated (Ryan et al., 1997). In the other hand, extrinsic motivation refers to engaging in an activity for instrumental reasons, such as rewards (Ryan and Deci, 2000a,b). For example, an aerobics practitioner who practices your activity for improving his appearance is said to be extrinsically motivated (Ryan et al., 1997).

In order to understand the motives for physical activity, Ryan et al. (1997) developed a scale named Motives for Physical Activity Measure-Revised (MPAM-R) where used as the theoretical background of the SDT. The scale was composed by 30 items divided into five factors: (1) Fitness; (2) Appearance; (3) Competence/Challenge; (4) Social; and (5) Enjoyment. In summary, the original scale showed acceptable psychometric properties by Exploratory Factor Analysis (EFA) and Cronbach’s alpha ranging between 0.78 to 0.92.

To the best of our knowledge, this scale was translated and cross-culturally adapted in three studies and two languages. Celis–Merchán (2006) performed the adaptation of the Spanish version of the MPAM-R on a sample of 120 Colombian subjects. Cronbach’s alpha of the Celis–Merchán (2006) study range between 0.75 to 0.91. In addition, the authors performed an EFA that showed several problems (Celis–Merchán, 2006). Murcia et al. (2007b) adapted MPAM-R for the Spanish population in a large sample size (464 subjects). The final version of the scale purpose by the Murcia et al. (2007b) was composed by the 28 items with acceptable results by the EFA. In addition, the Cronbach’s alpha range between 0.80 to 0.87. Finally, Gonçalves and Alchieri (2010) performed the cross-cultural adaptation and validation of the scale for Brazilian Portuguese version. The results showed that the 26 items were not acceptable using Confirmatory Factor Analysis (CFA) standards [χ^2^_(289)_ = 757.75; GFI = 0.83; AGFI = 0.80, and RMSE = 0.07], although the authors considered that the values were acceptable. In addition, Gonçalves and Alchieri (2010) results showed acceptable values of the Cronbach’s alpha (between 0.75 to 0.88). Therefore, results around MPAM-R Brazilian version suggest the importance of psychometric review.

Moreover, in all MPAM-R versions, the factor scores are calculated by the mean of scores by items of the factor (for example, mean of the scores of the items 6,15,21,28, and 30 for social factor in MPAM-R). Although, this method is largely used in Sports Psychology Scales (Lonsdale et al., 2008; Pelletier et al., 2013) there are several other methods (for more details, see DiStefano et al., 2009), that can be interesting. One option would be the weighted method (DiStefano et al., 2009), that used the factor loading extracted by the EFA or CFA. In the common method (mean of scores by items), all items on a factor are given equal weight, regardless of the factor loading value extracted in EFA or CFA. In summary, the common method does not involve item loading values, thereby disregarding the strength (or weight) for each item. Therefore, items with relatively low loading values are given the same weight in the factor score as items with higher loading values. On the another hand, in the weighted method, the factor score is based on factor loading extracted by EFA or CFA, to each item. Therefore, one advantage this method is that items with higher factor loadings have greater weight in the factor score, as items with lower factor loadings have less weight have less weight in the factor score (DiStefano et al., 2009).

Thus, the present study aims to perform a new translation and cultural adaptation of the MPAM-R for the Portuguese languages and analyze its psychometric properties. In addition, verify the agreement between common (mean by items) and weighted methods to calculate factor scores.

## Materials and Methods

### Cross-Cultural Adaptation

**FIGURE 1:**
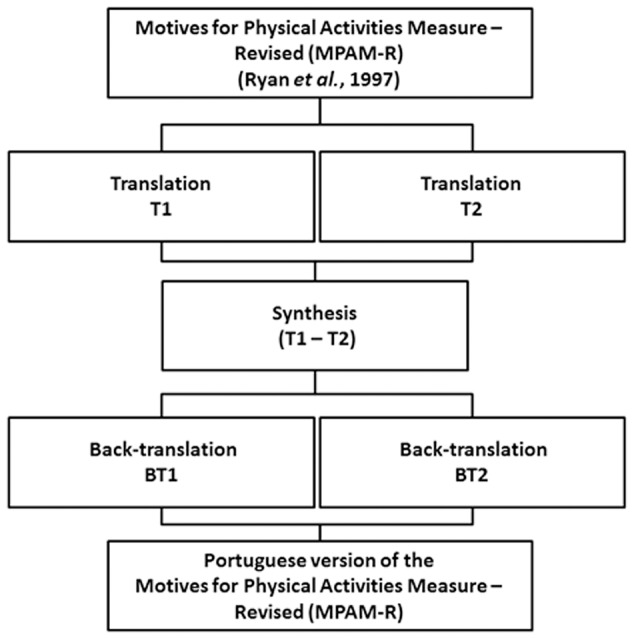
**Summary of the cross-cultural adaptation method**.

Initially, we contacted the researcher responsible for the scale to request his authorization to conduct the MPAM-R scale translation. After his approval, we started the translation process. The cross-cultural adaptation (**Figure 1**) began with a translation of the original scale of the MPAM-R into the Portuguese language. This step was carried out by two translators were native Portuguese speakers and had fluent English. The translations were independent, with two Portuguese versions (T1 and T2) of the scale being produced. A group of two Ph.D.’s with experience in Cross-Cultural Adaptation compared the different translations and evaluated semantic discrepancies, including any linguistic or conceptual issues. Secondly, a synthesis of the translations was obtained. The synthesis (T1 – T2) was independently back-translated to English (BT1 and BT2) by two translators who have English as their mother tongue. The same group of two Ph.D.’s. merged the two back translations into the final Portuguese version of MPAM-R (P-MPAM-R).

### Validation

#### Sample

The final stage of adaptation process suggested by Beaton et al. (2000) is the pre-test. According to Beaton et al. (2000), the recommend sample size for this stage should range from 30 and 40 individuals. We increased this suggestion for a convenience sample of 300 individuals attending four different gyms (more details in **Table 1**) with a mean of age of 28.37 (±7.11) years, who are enrolled in physical activities programs for at least 6 months (mean of regular physical activity practices of 51.60 ± 28.82 months). The sample size was increased in order to perform more robust statistical analysis (e.g., CFA and/or EFA). In addition, our sample size is in accordance with most used recommendation (Brown, 2006; Kline, 2011) for EFA and CFA, which is a ratio of 10:1 (ratio of the number of the subjects *per* number of items). The study was approved by the ethical committee of the Universidade Federal de Viçosa, and participants signed an informed consent after receiving a full explanation of the study.

**Table 1:** Sample characteristics.

**Sex**	
Male	129 (43%)
Female	171 (57%)
**Education level**	
Undergraduate	99 (33%)
Graduated	201 (67%)
**Marital status**	
Married/living with partner	102 (44%)
Single/divorced/widowed	198 (66%)
**Types of activity**	
Weight training	186 (62%)
Swimming	45 (15%)
Dancing	39 (13%)
Martial Arts	30 (10%)

### Procedures

At the beginning, contact was made with the manager of the gyms in which all the research objectives and procedures were presented. Afterward, the volunteers were personally invited to participate in the study. All volunteers were informed of the objectives and all procedures of the study, and were informed that would not receive any financial benefit from participation. All those who were willing to participate and had enough time available to respond to the instrument were selected. The scale was applied individually in the appropriate room (comfortable chair and table; without noise), before the regular physical activity practice.

### Instrument

The original Motivation for Physical Activities Measure-Revised (MPAM-R) is composed by 30 items in five general motives for physical (factors): Enjoyment (seven items); Competence (seven items); Appearance (six items), Fitness (five items), and Social (five items). Items should be responded in 7-point Likert scale (1 – “not at all true for me” to 7 – “very true for me”) (Ryan et al., 1997).

### Statistical Analysis

Kaiser–Meyer–Olkin (KMO) measure of sampling adequacy and Bartlett’s test of sphericity were used for the evaluation of model sufficiency (Field, 2009). High values of KMO (more than 0.70) generally indicate that a factor analysis may be useful with the data (Field, 2009). Bartlett’s test of sphericity tests the hypothesis that a correlation matrix is an identity matrix, which would indicate that variables are unrelated and therefore unsuitable for structure detection (Field, 2009). Values lower than 0.05 of significance probability indicate a satisfactory factor analysis. CFA and EFA were conducted in order to assess the model fit of the original model. Weighted least squared method (WLSMV) estimator was used since it recommended as a good alternative when data are non-normal due to the ordinal nature (e.g., a Likert-type scale of fewer than seven points) of the scale (Chen et al., 2015).

As suggested by some authors (e.g., Brown, 2006; Kline, 2011), Root Mean Square Error of Approximation (RMSEA), Comparative Fit Index (CFI), Tucker-Lewis Index (TLI), and Standardized Root Mean Square Residual (SRMR) were used to evaluate the model fit. In addition, to minimize the impact of sample size on the model we examined the Relative Chi-Square (χ2/df). The Relative Chi-Square is a measure to evaluate overall model fit; a value as low as 2.0 was recommended for a good model fit. A cut-off criterion equal or higher than 0.90 was recommended for the CFI and TLI. In addition, RMSEA and SRMR values less than 0.08 are considered acceptable. EFA using Geomin oblique rotation method was conducted, when necessary. In addition, instrument’s internal reliability (Cronbach’s alpha α ≥ 0.70) was computed.

In order to calculate factor scores, we adapt the weighted method (DiStefano et al., 2009). Firstly, sums of the factor loadings are calculated. Secondly, item’s factor loadings are standardized by the sum of factor loadings. Then, factor score is computed by the sum of each item score by multiplying the standardized factor loading by the score of the item. For instance, in a five items factor (Factor loading – Item 1 = 0.80; Item 2 = 0.60; Item 3 = 0.40; Item 4 = 0.60; Item 2 = 0.80), the sum of the factor loading is 3.20 (Σ of the factor loading of the item 1 to 5). The standardized factor loading of the items are: Item 1 – 0.80/3.20 = 0.25; Item 2 – 0.60/3.20 = 0.19; Item 3 – 0.40/3.20 = 0.13; Item 4 – 0.60/3.20 = 0.19; Item 5 – 0.80/3.20 = 0.25. Assuming this hypothetical example that subject one scored 7 (“Very true for me”) on all items, the factor score of the subject is 7 [(7^∗^0.25)+(7^∗^0.19)+(7^∗^0.13)+(7^∗^0.19)+(7^∗^0.25)]. Finally, to verify agreement between factors scores by weighted method and by common method (mean of the scores), we used Bland and Altman plots (Bland and Altman, 1999) and Paired *t*-test (Field, 2009).

## Results

### Cross-Cultural Adaptation

The original and translated Portuguese versions of the MPAM-R are showed in **Table 2**.

**Table 2:** Translation of the Motives for Physical Activity Measure Revised (MPAM-R).

	Original version	Portuguese version
1.	Because I want to be physically fit.	Por que eu quero ficar fisicamente em forma.
2.	Because it’s fun.	Por que é prazeroso.
3.	Because I like engaging in activities which physically challenge me.	Por que gosto de praticar atividades fisicamente desafiadoras.
4.	Because I want to obtain new skills.	Por que quero aprender novas habilidades.
5.	Because I want to look or maintain weight so I look better.	Por que quero perder ou manter o peso e ter uma melhor aparência.
6.	Because I want to be with my friends.	Por que quero encontrar meus amigos.
7.	Because I like to do this activity.	Por que gosto de praticar essa atividade.
8.	Because I want to improve existing skills.	Por que quero melhorar as habilidades que já tenho.
9.	Because I like the challenge.	Por que gosto do desafio.
10.	Because I want to define my muscles so I look better.	Por que quero definir meus músculos e ter uma melhor aparência.
11.	Because it makes me happy.	Por que fico feliz.
12.	Because I want to keep up my current skill level.	Por que quero manter meu nível de habilidade atual.
13.	Because I want to have more energy.	Por que quero ter mais energia.
14.	Because I like activities which are physically challenging.	Por que gosto de atividades fisicamente desafiadoras.
15.	Because I like to be with others who are interested in this activity.	Por que gosto da companhia de outras pessoas interessadas nessa atividade.
16.	Because I want to improve my cardiovascular fitness.	Por que quero melhorar minha condição cardiovascular.
17.	Because I want to improve my appearance.	Por que quero melhorar a minha aparência.
18.	Because I think it’s interesting.	Por que acho que é interessante.
19.	Because I want to maintain my physical strength to live a healthy life.	Por que quero manter minha força física para levar uma vida saudável.
20.	Because I want to be attractive to others.	Por que quero que os outros me achem atraente.
21.	Because I want to meet new people.	Por que quero conhecer novas pessoas.
22.	Because I enjoy this activity.	Por que gosto dessa atividade.
23.	Because I want to maintain my physical health and well-being.	Por que quero manter minha saúde física e bem-estar.
24	Because I want to improve my body shape.	Por que quero melhorar a forma de meu corpo.
25.	Because I want to get better at my activity.	Por que quero melhorar na minha atividade.
26.	Because I find this activity stimulating.	Por que acho essa atividade estimulante.
27.	Because I will feel physically unattractive if I don’t.	Por que me acho fisicamente feio se não o fizer.
28.	Because my friends want me to.	Por que meus amigos querem que eu o pratique.
29.	Because I like the excitement of participation.	Por que gosto do prazer de participar.
30.	Because I enjoy spending time with others doing this activity.	Por que gosto de passar tempo com outras pessoas praticando essa atividade.

### Psychometric Properties

Firstly, we analyzes descriptive data through the KMO measure of sampling adequacy (KMO = 0.925) and the Barlett’s test of sphericity <0.001.

Then, the Cronbach’s alpha was conducted to the initial proposal of 30 items, in which all dimensions showed values higher than 0.70. Despite favorable results of Cronbach’s alpha (**Table 3**), the CFA of the original model (30 items) demonstrated that the model fit index measures were not satisfactory (CFI = 0.89; TLI = 0.87; RMSEA = 0.103 e; χ^2^ = 1661.165 e gl = 395; *p* < 0.001).

**Table 3:** Cronbach’s alpha of the 30 and 26 items versions.

	30 items	26 items
Enjoyment	0.92	0.92
Competence	0.88	0.86
Appearance	0.76	0.76
Fitness	0.79	0.80
Social	0.83	0.83

**FIGURE 2:**
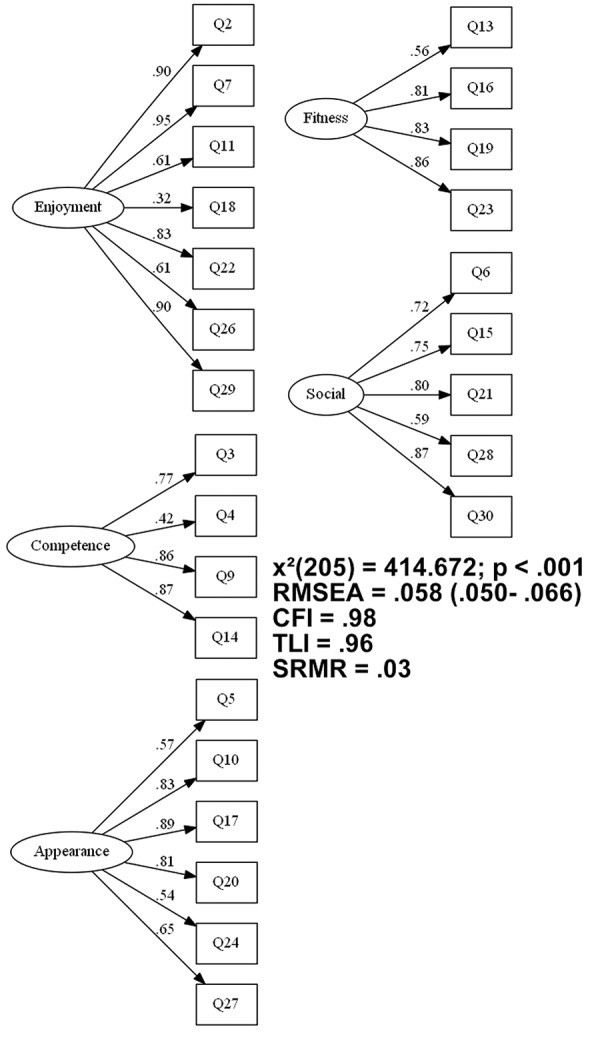
**Approximate fit index measures and loadings of the final model**.

As the model fit index measures of the original model were not satisfactory, an EFA with five factors was made. Those analysis results showed positive approximate fit index (CFI = 0.98; TLI = 0.96; RMSEA = 0.056 e; χ^2^ = 567.813 and gl = 295; *p* < 0.001). However, some items carried in dimensions that were not conceptually appropriate and items with factor loading lower than 0.30 were found. Therefore, the items with loading higher than 0.30 in two dimensions (8 and 25) or with factor loading lower than 0.30 in dimensions that were conceptually considered in the original model (1 and 12) were removed from the next analysis. After the withdrawal of the four items, the final model (**Figure 2**) came up with acceptable fit index (CFI = 0.98; TLI = 0.96; RMSEA = 0.058 e; χ^2^ = 414.672 e gl = 205; *p* < 0.001. Furthermore, all values of Cronbach’s alpha were higher than 0.70 in all dimensions (**Table 3**).

### Agreement between of Factor Scores

Factor scores formula extracted by the factor loading (**Figure 2**) of the EFA of MPAR-R’s final version to calculate the factor scores by weighted method are shown below:

• Enjoyment = [(Q2^∗^0.18) + (Q7^∗^0.18) + (Q11^∗^0.12) + (Q18^∗^0.06) + (Q22^∗^0.16) + (Q26^∗^0.12) + (Q29^∗^0.18)];
• Competence = [(Q3^∗^0.26) + (Q4^∗^0.14) + (Q9^∗^0.30) + (Q14^∗^0.30)];
• Appearance = [(Q5^∗^0.13) + (Q10^∗^0.19) + (Q17^∗^0.21) + (Q20^∗^0.19) + (Q24^∗^0.13) + (Q27^∗^0.15)];
• Fitness = [(Q13^∗^0.18) + (Q16^∗^0.27) + (Q19^∗^0.27) + (Q23^∗^0.28)]
• Social = [(Q6^∗^0.19) + (Q15^∗^0.20) + (Q21^∗^0.22) + Q28^∗^0.16) + (Q30^∗^0.23)]

**FIGURE 3:**
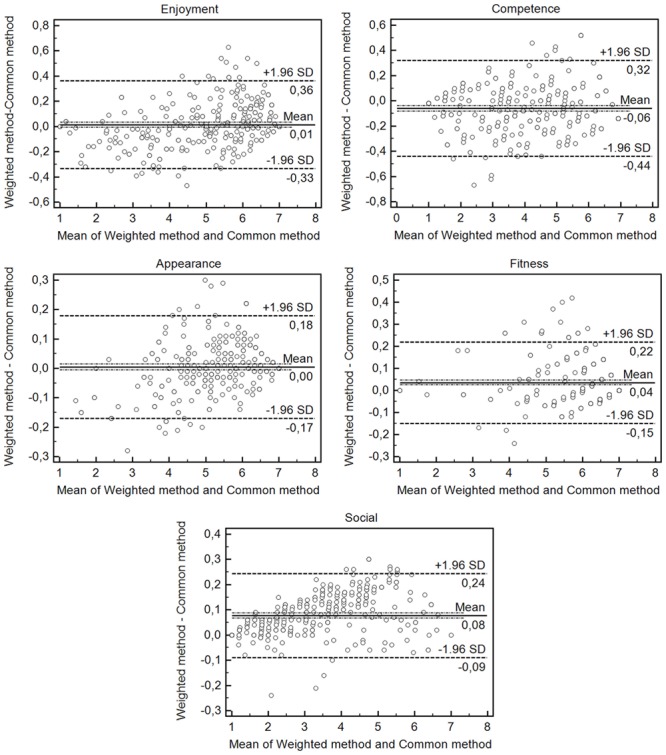
**Bland-Altman plots**. The dashed bold lines represent the mean difference score. The dashed lines represent the limits of agreement (mean ± 1.96 × the standard deviation of the difference score).

Bland and Altman plots of data from weighted and common methods (mean of the item scores) are showed in **Figure 3**. In “Enjoyment” [mean bias of 0.01 lower (-0.33) and upper (0.36) 95% confidence interval] and “Appearance” [mean bias of 0.00 lower (-0.17) and upper (0.18) 95% confidence interval] factors produced a lower mean bias than others factors when agreements between weighted and common (mean of the item scores) methods are analyzed. In addition, **Table 4** showed analysis by Paired *t*-test that did not show significant differences in “Enjoyment” [*t*(299) = 1.33; *p* = 0.184] and “Appearance” [*t*(299) = 0.83; *p* = 0.409] factors scores between weighted and common (mean of the item scores) methods. All others comparisons were significant (*p* < 0.001).

**Table 4:** Comparison between weighted methods and common method.

Factor	Weighted method (*n* = 300)		Common method (*n* = 300)		*P*-value
	Mean	*SD*	Mean	*SD*	
Enjoyment	5.15	1.56	5.13	1.50	0.184
Competence	3.86	1.70	3.92	1.69	**<0.001^∗^**
Appearance	5.30	1.20	5.29	1.17	0.409
Fitness	6.11	1.11	6.07	1.11	**<0.001^∗^**
Social	3.32	1.53	3.24	1.49	**<0.001^∗^**

### Normative Data

**Table 5** shows normative data for the Portuguese version of MPAR-R.

**Table 5:** Interpretative parameters of the MPAM-R for Weighted and Common Methods.

	Weighted method (*n* = 300)					Common method (*n* = 300)				
	En	Co	Ap	Fi	So	En	Co	Ap	Fi	So
Mean	5.14	3.86	5.30	6.11	3.32	5.13	3.92	5.29	6.07	3.24
*SD*	1.56	1.70	1.20	1.10	1.53	1.50	1.69	1.17	1.11	1.49
Minimum	1.00	1.00	1.00	1.00	1.00	1.00	1.00	1.50	1.00	1.00
Maximum	7.00	6.86	7.00	7.00	7.00	7.00	7.00	7.00	7.00	7.00
Percentile 5	2.18	1.00	3.12	4.01	1.00	2.15	1.00	3.17	3.76	1.00
Percentile 25	4.06	2.53	4.53	5.74	2.00	4.03	2.56	4.50	5.75	2.00
Percentile 50	5.56	3.85	5.40	6.46	3.27	5.57	4.00	5.33	6.50	3.20
Percentile 75	6.40	5.19	6.25	7.00	4.54	6.28	5.25	6.17	7.00	4.40
Percentile 95	7.00	6.72	7.00	7.00	6.01	7.00	6.75	7.00	7.00	5.99
Percentile 99	7.00	6.86	7.00	7.00	6.62	7.00	7.00	7.00	7.00	6.59

*SD, standard deviation; En, enjoyment; Co, competence; Ap, appearance; Fi, fitness; So, social.*

## Discussion

The aim of this study was to perform a cultural adaptation of the MPAM-R for the Portuguese languages, analyze its psychometric properties and compare the factor loadings calculated by mean and by weighted method. In summary, although the Portuguese version of the MPAM-R has fewer items when compared with the original version (Ryan et al., 1997), our version had acceptable psychometric proprieties and internal consistency. In addition, our results showed that the factor score calculated by mean and by the weighted method are different in three of the five factors.

In general, most of the sports psychology scales were developed in English-speaking countries, so cross-cultural and international collaborative studies, as well as the possibility of testing theories are needed. Thus, researchers need reliable and valid instruments in other languages. Nowadays, there are well-established methodological approaches for translating, adapting and validating instruments (Beaton et al., 2000; Sperber, 2004). Although, there was no clear consensus on how each method should be used. To the best of our knowledge, the MPAM-R was adapted to Spanish (Celis–Merchán, 2006; Murcia et al., 2007b) and Portuguese (Gonçalves and Alchieri, 2010). In general, the translation process used by those of studies was quite mixed, being more (Gonçalves and Alchieri, 2010) or less rigorous (Celis–Merchán, 2006). Thus, ours and Gonçalves and Alchieri (2010) studies have used the most rigorous approaches for translating the MPAM-R.

EFA and CFA are widely used multivariate statistical procedures that serve as tool to scale validation. EFA was used in Spanish versions (Celis–Merchán, 2006; Murcia et al., 2007b). However, EFA in Celis–Merchán (2006) study showed many problems. Instead, EFA showed acceptable results in the final version of the scale purpose by the Murcia et al. (2007b). Although the use of EFA is an interesting and useful method in scales validation, it is important to note that none of the Spanish studies performed an assessment of model fit. Model fit is obtained through the several statistical tests used to determine how well the model fits to the data (Brown, 2006; Kline, 2011) and it is considered a robust statistical technique to validate scales. Unlike the Spanish versions (Celis–Merchán, 2006; Murcia et al., 2007b), Gonçalves and Alchieri (2010) performed a CFA and reported model fit. However, using the recommendations indicated by some authors (Brown, 2006; Kline, 2011), the results showed that model fit was not acceptable [χ^2^_(289)_ = 757.75; GFI = 0.83; AGFI = 0.80, and RMSE = 0.07], although the authors considered acceptable. A similar model fit as also found in another study (Wilson et al., 2002) that aimed to verify the psychometric properties of the MPAM-R. Meanwhile the authors considered that the values were not acceptable. Thus, our study used rigorous statistical approaches and the final version of the MPAM-R indicated that all parameters recommended for an excellent model fit were found [*X*^2^(205) = 414.672; *p* < 0.001; CFI = 0.98; TLI = 0.97; RMSEA = 0.058; SRMR = 0.03].

Reducing the number of items is a common practice among translation and cross-cultural adaptation of scales. Especially in MPAM-R, Murcia et al.’s (2007b) and Gonçalves and Alchieri’s (2010) studies had final versions with 28 and 26 items, respectively. In the present study, two items were excluded due to factor loading higher than 0.30 in two dimensions (8: “to improve existing skills” and 25: “to get better at activity”); and other two items were excluded because showed factor loading lower than 0.30 in dimension that were conceptually contemplated by the original model (1: “want to be physically fit” and 12: “to keep up current skill level”). Although the P-MPAM-R has a smaller number of items than the original scale, the psychometric properties showed good values. Furthermore, the smaller the scale is, the shorter will be the time for your application, which ease data collection process. Moreover, as in other studies (Celis–Merchán, 2006; Murcia et al., 2007b; Gonçalves and Alchieri, 2010), the final version of the scale presented the same structure of five factors (enjoying, competence, appearance, fitness, and social) found in the original study (Ryan et al., 1997).

Regarding reliability, Cronbach’s alpha is a generalization of the internal consistency reliability coefficient (Field, 2009). As can be seen in **Table 6** all versions (Ryan et al., 1997; Murcia et al., 2007b; Gonçalves and Alchieri, 2010), including the present study, showed higher values than recommended (>0.70).

**Table 6:** Cronbach’s alpha Index to the MPAM-R in different studies.

Studies	Cronbach’s alpha index by dimensions				
	Enjoyment	Competence	Appearance	Fitness	Social
Ryan et al., 1997	0.92	0.88	0.91	0.78	0.83
Celis–Merchán, 2006	0.75	0.87	0.90	0.81	0.75
Murcia et al., 2007b	0.84	0.85	0.87	0.80	0.81
Gonçalves and Alchieri, 2010	0.88	0.75	0.79	0.84	0.85
Present study	0.92	0.86	0.76	0.80	0.83

In the Brazilian context, other scales can be used to investigate the motives to physical activity, since that the most used is IMPRAFE (Motivation to Regular Physical Activity Inventory (in Portuguese “Inventário de Motivação para a Prática de Atividade Física”) (Barbosa, 2006). The scales were composed by some factors named: stress control, social, competence, appearance, enjoyment and fitness. Barbosa (2006) conducted the validation process of the IMPRAFE with 126 items and its short version with 48 items. As in Gonçalves and Alchieri (2010) study the results showed that in 120 items version of the IMPRAFE, the model fit was not acceptable [GFI = 0.859, AGFI = 0.854 and RMS = 0.065]. However, the short version with 48 items showed satisfactory [GFI = 0.952, AGFI = 0.948 and RMS = 0.058]. In both versions, the IMPRAFE showed Cronbach’s alpha higher than 0.70. In summary, the IMPRAFE-short version (Barbosa, 2006) can be an excellent scale to investigate Motivation for Physical Activity in a Brazilian context. However, the fact that was not translated or adapted to other contexts (for example, English) does not allow cross-cultural and international collaborative studies. In addition, the fewer of items of MPAM-R reduces application time to IMPRAFE, which facilitate data collection process.

Overall, the present study compared two ways to calculate the factor score. In general, factor scores are calculated by the mean of scores by items of the factor. Therefore, when performing this procedure it is suggested that all items have the same weight for factor calculation. However, in EFA or CFA, the factor loadings show that some items load more than others in the same latent variable. For this reason, we believe that the current way factor scores are calculated needs to be reviewed, though being largely used. In the present study, we used an adaptation of the weighted method (DiStefano et al., 2009), that used the factor loading extracted by the EFA to create weights for each item of the factor. Our results showed that when compared two methods to calculate factors scores, only “Enjoyment” and “Appearance” factors showed agreement between methods to calculate factor scores. On the other hand, Paired *t*-test found that all others comparisons were significant (*p* < 0.001). Hence, values calculated by the two methods exhibit statistical significant differences. In addition, disagreements (see **Figure 3**) of this factors (“Competence”; “Fitness”; and “Social”) do not present a pattern (e.g., overestimation or underestimation the values). Thus, it is possible to consider that the theoretical changes in the way of calculating the factor score modified the results. Basically, we considered that the weighted method proposed in the present study presents a more appropriate theoretical background. For this reason, we believe that the weighted method can be a more appropriate alternative to calculating factor scores. Although even more refined methods exist (for more details, see DiStefano et al., 2009) we chose to adapt the weighted method because it enables the comparison between the factors since the units of measure are the same as the original scale (variation between 1 and 7). In conclusion, we believed that our not much common weighted method could be an alternative to calculating factor scores, once it takes into account the factor loading of the item extracted by the Factorial Analysis.

Our study suffers from some limitations. Firstly, reliability, in a practical definition, is the ability of an instrument to measure consistently (Hopkins, 2000), and it is closely associated with its validity (Tavakol and Dennick, 2011). In the present study, we used the Cronbach’s alpha as the reliability measure, however, this measure has received a lot of criticism (Sijtsma, 2009). Although understanding the limitations to the Cronbach’s alpha (Sijtsma, 2009), and that other methods could have been used (Hopkins, 2000), we chose it because it is probably the most used reliability measure in scales validation, as well as, all other studies (Celis–Merchán, 2006; Murcia et al., 2007b; Gonçalves and Alchieri, 2010), including the original scale (Ryan et al., 1997), also used this reliability measure. In the end, it was not possible to test our final model proposed by the EFA with a new CFA in a different and large sample, however, this may be a proposal for further study.

## Conclusion

The present study provides evidence of validity and excellent psychometrics properties of the Portuguese version of the MPAM-R in which 7-items measure enjoyment, 4-items measure competence, 6-items measure appearance, 4-items measure fitness, and 5-items measure social. Therefore, the Portuguese version of the MPAM-R may be used appropriately and successfully to measure the motives for physical activity. In addition, we showed another way to calculate factor score named weighted method that is different from the common method (mean of the score), but can be a more appropriate alternative to calculate factor scores.

## Author Contributions

MA, ML, JdP, and VdC designed the study and analyzed data. LF collected the data. All authors participated in the interpretation of the data. MA drafted the manuscript. All authors helped with the writing of the manuscript. All authors approved the final version of the manuscript submitted for publication.

## Conflict of Interest Statement

The authors declare that the research was conducted in the absence of any commercial or financial relationships that could be construed as a potential conflict of interest.
